# Botulinum toxin in speech rehabilitation with voice prosthesis after total laryngectomy

**DOI:** 10.1016/S1808-8694(15)31093-4

**Published:** 2015-10-19

**Authors:** Carlos Takahiro Chone, Cristiane Teixeira, Nelson A. Andreollo, Ana Lucia Spina, Irene H.K. Barcelllos, Elizabeth Quagliato, Agricio N. Crespo

**Affiliations:** 1 Doctor in medicine, specializing in Otorhinolaryngology, Medical School of the Campinas State University. Adjunct Professor / Otorhinolaryngology and Head & Neck Discipline / Unicamp; 2 Otorhinolaryngologist, medical resident / Otorhinolaryngology and Head & Neck Discipline / Unicamp; 3 Adjunct professor, Gastric Surgery Discipline / Gastrocentro / Unicamp; 4 Doctoral student, Otorhinolaryngology and Head & Neck Discipline / Unicamp. Speech therapist/ Otorhinolaryngology and Head & Neck Discipline / Unicamp; 5 Adjunct professor / Radiology Department / Unicamp; 6 Adjunct professor / Neurology Departmento / Unicamp; 7 Adjunct professor / Otorhinolaryngology and Head & Neck Discipline / Unicamp. Chief of the Otorhinolaryngology and Head & Neck Discipline / Unicamp. Otorhinolaryngology and Head & Neck Discipline / Unicamp

**Keywords:** laryngeal cancer, total laryngectomy, botulinum toxin, voice, head and neck, tracheoesophageal speech

## Abstract

In tracheo esophageal puncture (TEP), we carry out a myotomy of the pharynx constrictor muscle; however, about 9 to 79% of patients need such procedure. The consequence of such procedure is an increase in salivary fistula rates in the postoperative. Botulin toxin is used in an outpatient basis.

**Aim:**

analyzing the efficacy of botulin toxin (BT) use in the rehabilitation of totally laryngectomized patients with tracheoesophageal voice (TEV) with spasms (S) of the pharyngoesophageal segment (PES) without myotomy.

**Materials and Methods:**

We analyzed eight patients submitted to total laryngectomy (TL), rehabilitated with TEV, with speech prosthesis (SP) and struggle to utter voice because of PES spasms. They were all submitted to treatment of such motor alteration with the injection of 100 units of BT in the PES. The evaluation was based on perceptive voice analysis, video fluoroscopy (VF) of the PES, acoustic voice analysis and computerized manometry (CM) of the PES, all before and after BT injection.

**Study design:**

prospective

**Results:**

There was a reduction in PES CM pressure after BT injection. Acoustic analysis showed an improvement in harmonics quality after treatment. There was smoother voice utterance and spasm improvement after BT.

**Conclusion:**

all patients with PES spasms presented vocal improvement after BT injection in the PES.

## INTRODUCTION

About 9% to 79% of total laryngectomy (TL) patients undergoing rehabilitation with tracheoesophageal voice (TEV), phonatory prostheses (PP), and primary or secondary tracheoesophageal needle puncture (TEP), present effort-induced phonatory difficulties associated with motility alterations in the pharyngoesophageal segment (PES) secondary to spasm of this segment.^1^, ^2^, ^3^, ^4^, ^5^, ^6^, ^7^, ^8^, ^9^, ^10^, ^11^, ^12^, ^13^ There are three approaches for the treatment of PES alterations: myotomy of the middle and lower constrictors of the pharynx, neurectomy of the pharyngeal plexus, and the recently described chemical denervation of the PES with the botulinum toxin (BT).^6^^,^^7^^,^^8^^,^^10^^,^^11^^,^^14^, ^15^, ^16^, ^17^, ^18^, ^19^, ^20^, ^21^, ^22^, ^23^ The BT is a presynaptic blocker of acetylcholine release at the neuromuscular junction. Videofluoroscopy^3^^,^^4^^,^^7^^,^^15^^,^^24^ and computed manometry (CM) demonstrate relaxation of the PES following the use of BT in this region. There are indirect assessment methods of the PES pressure, such as the modified esophageal insufflation test,^4^^,^^6^^,^^15^ measurement of intratracheal pressure and the phonation time.^7^^,^^18^ The purpose of this study was to assess the efficacy of the BT in TL patients rehabilitated by TEV with PP that presented emission of voice under effort due to spasm of the PES.

## MATERIAL AND METHOD

Eight TL patients rehabilitated by TEV with PP that presented excessive effort for voice emission and an almost absent maximum phonation time were selected from 71 TL patients that were similarly rehabilitated. All patients used an indwelling Blom-Singer (Inhealth®) PP, inserted after primary or secondary TEP.

The Research Ethics Committee of the local institution approved this study (protocol number 546/2005). All patients signed a free informed consent form.

**Figure 1:**
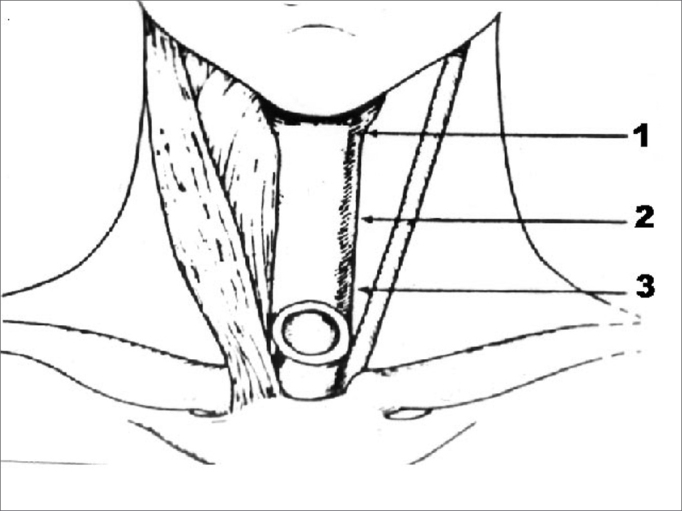
Representation of botulinum toxin injection areas in the three thirds of the pharyngoesophageal segment.

Testing was composed of perceptive voice analysis, measurement of the mean phonation time (mean of three consecutive measurements using a professional chronometer), swallowing and phonation testing by videofluoroscopy, four-channel CM, and computed acoustic voice analysis before and after injecting 100U of BT (Botox®) in the site of pharyngospasm. All patients also complained of mild dysphagia. BT was injected in each third of the PES (Figure 1) under electromyographic control of the pharyngeal constrictor muscles.

## RESULTS

**Figure 2:**
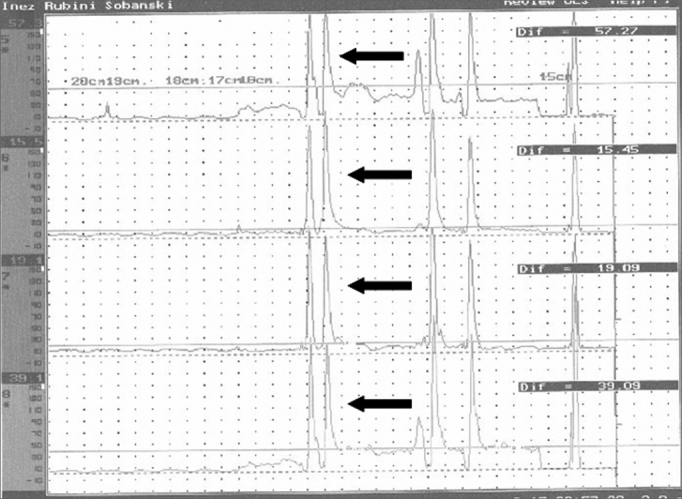

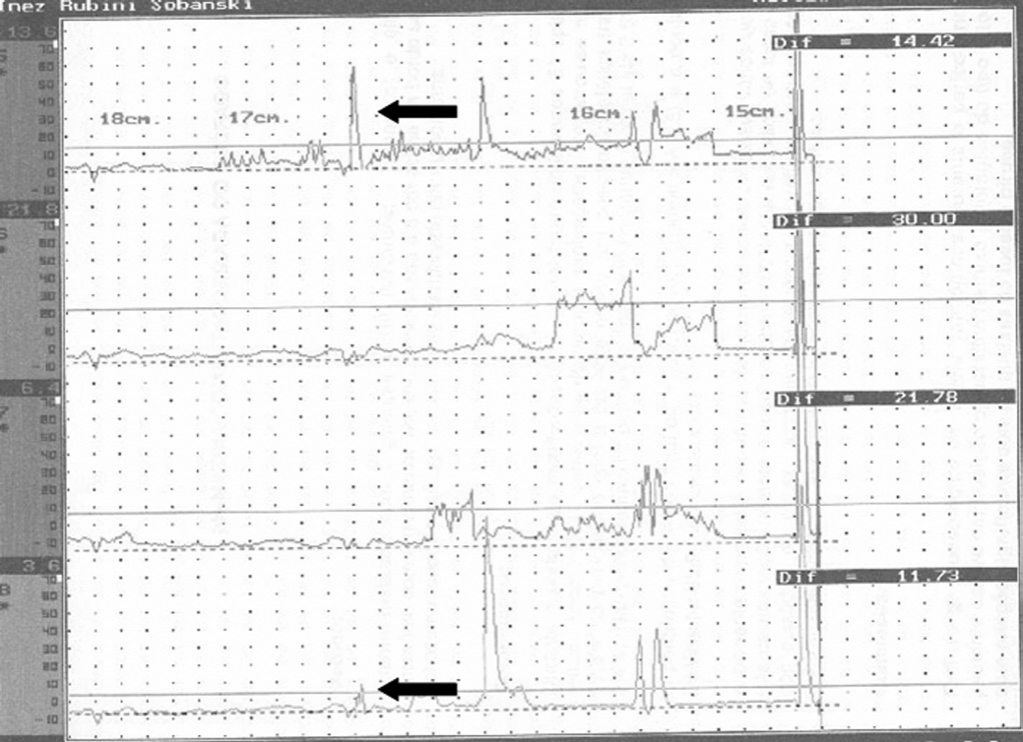
Pressures in the pharyngoesophageal segment for each computed manometry channel before (a) and after (b) application of the botulinum toxin. Each baseline is one channel. Arrows on each baseline show the pharyngoesophageal pressure.

CM revealed a decrease in the mean PES pressure following BT injection in eight patients (Table 1, Figure 2a and 2b).

**Table 1:** Pharyngoesophageal segment pressure on computed manometry before (PRE) and after (POST) application of 100 U of the botulinum toxin.

PATIENT	PRE	POST
1	33,0 mmHg	12,2 mmHg
2	17,27 mmHg	12,50 mmHg
3	16,79 mmHg	13,71 mmHg
4	32,7 mm Hg	19,6 mmHg
5	30,0 mmHg	14,1 mmHg
6	16,5 mmHg	13,6 mmHg
7	23,1 mmHg	15,4 mmHg
8	33,5 mmHg	13,4 mmHg

**Figure 3:**
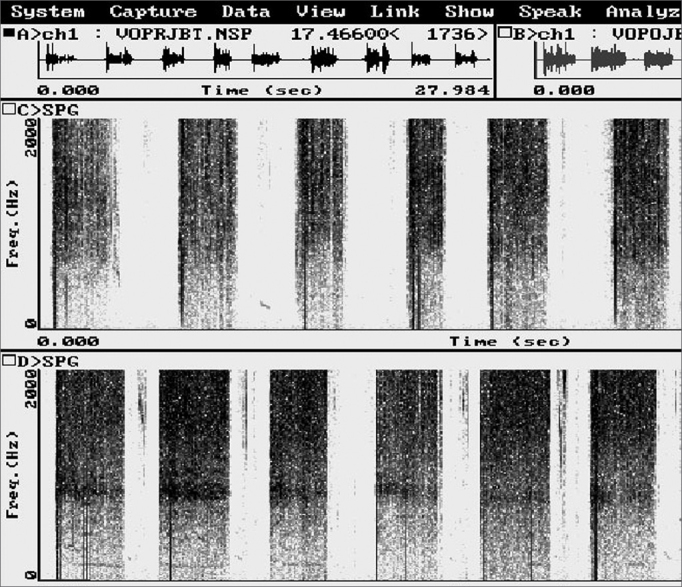

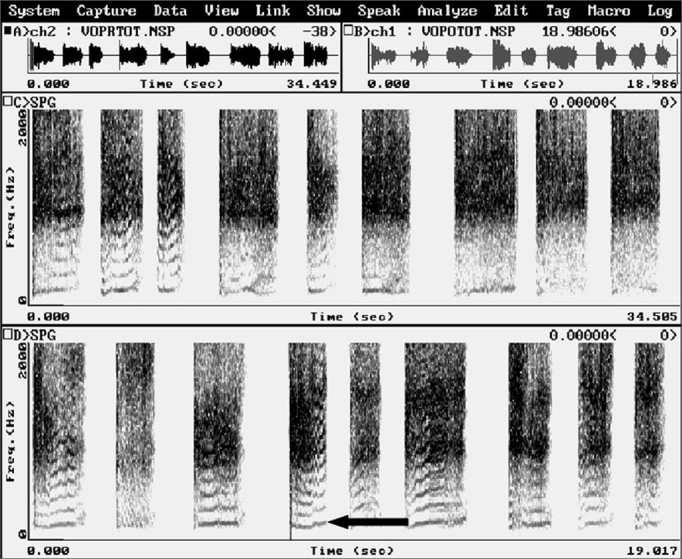
Computed acoustic analysis before (a) and after (b) injecting the botulinum toxin in the pharyngoesophageal segment. Harmonics were present after botulinum toxin injection (b), not visible before. An arrow shows a harmonic.

**Figure 4:**
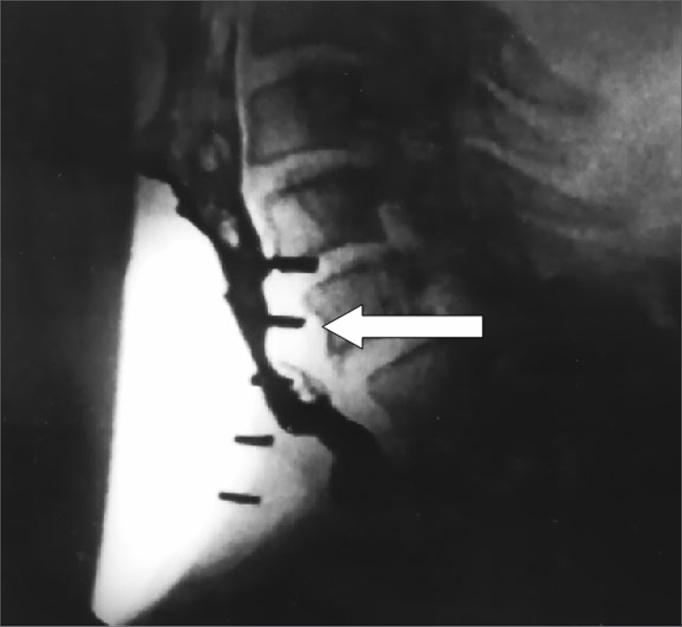

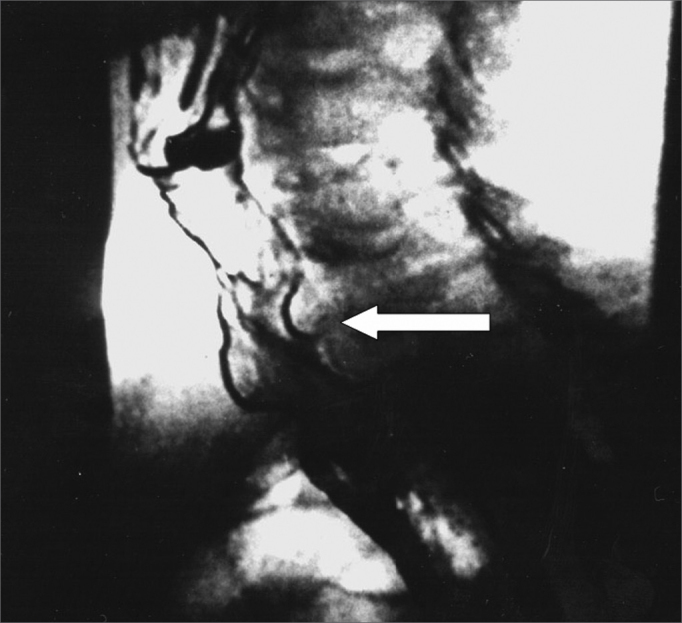
Videofluoroscopy - lateral view - during phonation before (a) and after (b) injection of the botulinum toxin in the pharyngoesophageal segment. The arrow shows spasm in the pharyngoesophageal segment before the injection (a) and absence of spasm after the injection (b), where increased antero-posterior distance may be perceived.

Computed acoustic analysis demonstrated the production of harmonics after BT injection in the PES of eight patients (Figure 3a and 3b). Harmonics were absent in these patients prior to therapy. Effortless voice production and increased phonation time became possible in these patients (Table 2). Before BT therapy, the phonation time in all patients was one second. All patients reported improved voice quality, as was demonstrated by the voice perception analysis. Videofluoroscopy of the PES during phonation showed that spasm of the PES decreased (Figure 4a and 4b). There were no adverse effects caused by the BT. There was clinical improvement of dysphagia in all patients.

**Table 2:** Mean phonation time values, in seconds, before (PRE) and after (POST) application of 100 units of the botulinum toxin in the pharyngoesophageal segment.

Patient		
Phonation time, in seconds	PRE	POST
1	1,0	9,0
2	1,0	7,0
3	1,0	7,0
4	1,0	7,5
5	1,0	8,0
6	1,0	8,5
7	1,0	6,8
8	1,0	7,0

## DISCUSSION

PES spasm is one of the causes of failure in the rehabilitation of TL patients with TEV and PP.^1^, ^2^, ^3^, ^4^, ^5^, ^6^, ^7^, ^8^, ^9^, ^10^, ^11^, ^12^, ^13^ Altered PES motor activity in these cases is a reflex initiated by air entering the esophagus, blocking air from progressing to the pharynx. Consequently, the pharyngeal mucosa does not vibrate, and there is no phonation.^1^^,^^3^, ^4^, ^5^^,^^7^, ^8^, ^9^^,^^13^^,^^18^ Spasm may be seen in videofluoroscopy done during phonation with the PP;^5^^,^^7^^,^^8^^,^^15^^,^^24^ it is absent during swallowing when the PES relaxes. During constriction there is no relaxation when swallowing. In this case, therapy is dilatation.^4^^,^^5^^,^^24^ These are natural protecting mechanisms against gastropharyngeal reflux; in TL patients, however, they become an obstacle against phonatory rehabilitation.^7^^,^^8^^,^^13^^,^^24^

BT injected in the PES was initially described in 1994^25^ for the treatment of swallowing disorders in which there was a hypertrophic or hypertonic upper esophageal sphincter. The dosages were 80 to 120 units. This method was first described for the treatment of PES spasm after TEP with PP in 1995.^15^ Some authors have shown effects lasting up to 2 years and 3 months with one initial dose only.^17^ A possible explanation for this is that after the first dose, patients adapt to the new condition;^17^ alternatively, denervation of the pharyngeal constrictor muscles due to presynaptic block by the BT might occur.

In primary TEP, myotomy of the middle and inferior pharyngeal constrictor muscles is one of the surgical steps.^10^^,^^26^ This step may be related to a higher incidence of postoperative salivary fistulae.^12^^,^^26^ In such cases, hospital stay and medical expenses are increased, and phonatory rehabilitation, oral feeding and even the beginning of postoperative radiotherapy are delayed. The true need for myotomy in TEP is a controversial issue in the literature, varying from 9% to 79% in TL patients.^1^, ^2^, ^3^, ^4^, ^5^, ^6^, ^7^, ^8^, ^9^, ^10^, ^11^, ^12^, ^13^ In secondary TEP, myotomy is associated with a 10% to 20% incidence of salivary fistulae,^26^ with the same consequences mentioned above. Using the BT for the treatment of PES spasm makes it possible to restrict myotomy for those patients that truly require this method for treating the PES; this avoids unnecessary procedures in the remaining patients, and reduces their surgery time and complication rate. Injection of the BT is done in an outpatient setting; the patient is seated and awake, and under electromyographic monitoring of the pharyngeal constrictor muscles. This procedure is less expensive than myotomy of the pharyngeal constrictor muscles.^17^ It should be borne in mind that even after myotomy of the middle and lower pharyngeal constrictor muscles, there may be spasm by reapproximation of muscle fibers.^1^^,^^7^^,^^10^^,^^11^^,^^17^ In such cases, the BT may also be used.

## CONCLUSION

The BT effectively decreased PES pressure in all patients, as assessed by CM.

All patients were able to produce harmonics following application of the BT in the PES, as demonstrated by computed acoustic voice analysis.

Phonation time was increased in all patients following application of the BT.

After injection of the BT, spasm of the PES decreased in all patients, as confirmed by videofluoroscopy.
